# A life cycle assessment of broiler chicken meat and egg production in Iceland

**DOI:** 10.1016/j.psj.2025.105072

**Published:** 2025-03-20

**Authors:** Sara Björg Guðjónsdóttir, Clara María Vásquez-Mejía, Sankalp Shrivastava, Ólafur Ögmundarson

**Affiliations:** a Faculty of Food Science and Nutrition, University of Iceland, Sæmundargata 12, 102 Reykjavík, Iceland; b Novo Nordisk Foundation Center for Biosustainability, Technical University of Denmark, Kgs. Lyngby 2800 Denmark

**Keywords:** Broiler chicken meat, Eggs, Life Cycle Assessment, Feed, Protein

## Abstract

As the world population grows, so does the production and consumption of emission-intensive foods. To reduce environmental impacts of food systems, significant and immediate changes are needed, both by adopting more environmentally sustainable practices and changing people's diets. To evaluate these strategies in connection with their potential environmental impacts, additional research is required on staple food systems. This study aims to assess the potential environmental impacts of broiler chicken meat and egg production in Iceland, which have become important protein sources among its inhabitants. Life Cycle Assessment (LCA) was used to evaluate and analyze possible environmental impacts. Although both products are derived from the same species, the common practice amongst farmers is to produce broiler chicken meat and eggs in separate housing systems, and thus, each system was modeled independently from each other. The system boundary for broiler chicken meat production was cradle-to-slaughterhouse gate with the functional unit (FU1) of 1 kg of broiler chicken (carcass weight). The life cycle stages included feed production, rearing of birds, manure management, and slaughterhouse for broiler chicken. In addition, a scenario analysis with the functional unit based on 100g of edible protein was also conducted to assess the impact for meat only. For egg production, the system boundary used was cradle-to-farm gate, with the functional unit (FU2) of 1 kg of eggs.

Results showed that feed production has the greatest environmental impact within the life cycle of both broiler chicken meat and eggs for the analyzed impact categories. Most of the impact was caused by wheat, soybean, and maize, all of which are commonly used in poultry feed at a global level. The results showed that Icelandic poultry production has lower environmental impact compared to other international studies. Furthermore, the outcome could be used when assessing the impacts of Icelandic dietary guidelines in the future.

## Graphical abstract

The environmental impacts of broiler chicken meat were estimated from the perspective of 1 kg carcass weight and 100 g of edible protein. This picture shows the global warming potential results when allocated to different chicken cuts based on their edible protein content.

**Figure:**
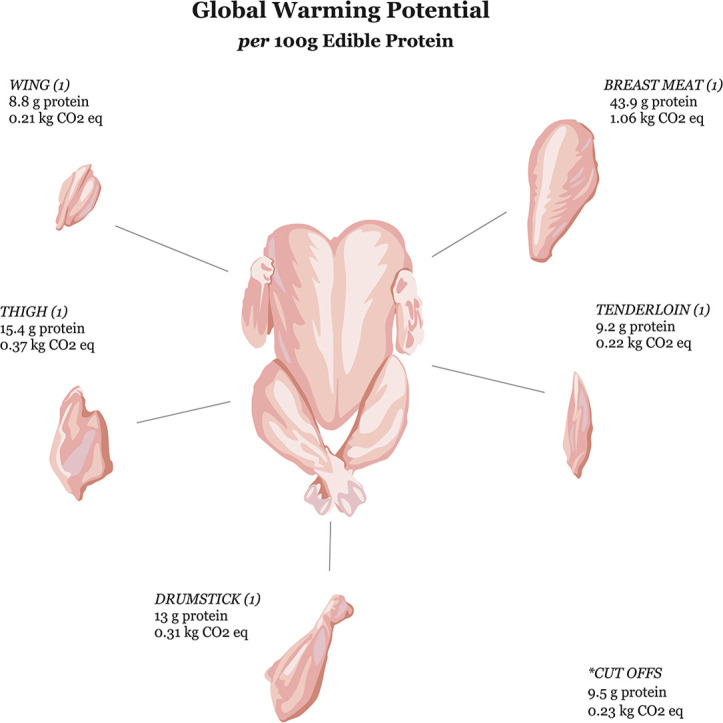

## Introduction

Since the mid-twentieth century, the world population has grown from 2.5 billion people in 1950 to 8 billion in 2022 and is predicted to further increase by an additional 1.7 billion people over the next 30 years (United Nations, 2022). To accommodate this growth, global food production is expected to increase by 35-56 % between 2010 and 2050 (Van Dijk et al., 2021).

The current food production format is unsustainable due to its numerous detrimental impacts on the environment, such as nutrient leaching caused by excessive use of fertilizers and pesticides, deforestation due to increased land requirements, and greenhouse gas (GHG) emissions caused by the use of fossil fuels, which in turn contributes to global warming (Conijn et al., 2018). Food production involves various interconnected stages that jointly form global food systems, which are estimated to be responsible for 23-42 % of global anthropogenic GHG emissions (IPCC, 2022), of which 12 % came from the livestock sector alone in 2015 (FAO, 2023). The global livestock sector has grown by 45 % in the last 20 years, reaching 337 million tonnes in production in the year 2020, where the vast majority was derived from three species: chicken (35 %), pig (33 %), and cattle (20 %) (FAO, 2022).

Moreover, 85 % of the total GHG emissions from livestock came from the same three animal species that dominate the production sector, but with different GHG contributions (cattle 62 %, pig 14 %, and chicken 9 %), with the largest impacts occurring through farming, generation of manure, energy consumption, and feed production (FAO, 2023; Cheng, 2022).

Food systems impact the environment significantly, as a result, the EAT-Lancet Commission has created scientific targets that aim to optimize human health and strive for sustainable food systems, combining planetary and health boundaries (Willett et al., 2019). These targets call for dietary shifts, where increased consumption of plant-based foods is vital, and the reduction of animal-based products, specifically red meat, is sorely needed (Willett et al., 2019). However, the EAT-Lancet also estimated that poultry consumption worldwide is within healthy planetary boundaries, meaning that it could potentially take a share of the red meat consumption which has surpassed the global health recommendations and environmental boundaries. While the EAT-Lancet framework has not been applied in Iceland, the latest national dietary survey showed that 11 % of the protein Icelanders consume comes from poultry, whereas 20 % comes from red meat (beef, lamb, pork, horse and reindeer) (Gunnarsdóttir et al., 2022).

Broiler chicken meat production has grown considerably in Iceland over the last 27 years due to a legal change in 1995 that allowed farmers to sell fresh/unfrozen poultry meat at supermarkets (Olgeirsson, 2003). Poultry production grew from 1,952 tonnes in 1995 to 9,501 tonnes in 2022, where 96.5 % of the production was chicken (breed Ross 308), and 3.5 % was turkey (Government of Iceland, 2023; Alifuglahald, 2024). Furthermore, broiler chicken meat outpaced the production of lamb for the first time in 2021, making it the most-produced meat in the country (Statistics Iceland, 2024a). Another important food system derived from chickens is egg production, which comes exclusively from the breeds Lohmann LSL and Lohmann LB (Alifuglahald, 2024). Egg production has slowly been growing from 3,000 tonnes produced in 1978 to 4,486 tonnes in 2022 (Statistics Iceland, 2024b). However, increased knowledge and technology has led to more efficient production, as fewer laying hens are now needed to provide the required number of eggs (Olgeirsson, 2003).

Assessing the environmental impacts of food systems can be challenging due to their complex supply chain interactions, geographical and temporal variations, and potential burden shifting (Sala et al., 2017). However, it has been found that Life Cycle Assessments (LCA) can yield estimates of environmental impact accurate enough to base decisions on (Blomhoff et al., 2023). While international LCA studies of food systems and food items are available, they might not necessarily represent the Icelandic reality as the country's food production runs almost exclusively on renewable energy for heating and electricity purposes, reducing fossil fuel emissions (Government of Iceland, 2024). For this reason, applying LCA to the production of staple food groups in Iceland could support the development of dietary guidelines that also take environmental impacts into consideration (Blomhoff et al., 2023).

The objective of this study was to estimate the environmental impacts of broiler chicken meat production, and egg production in Iceland. The research questions were:

1) What are the environmental impacts and environmental hotspots of producing eggs and broiler chicken meat in Iceland?
2) How do the potential environmental impacts of poultry production in Iceland compare to other countries?
3) What are the environmental impacts of 100 g of edible protein from eggs and broiler chicken meat?

There is currently no published peer reviewed LCA for Icelandic poultry or egg production, and only a handful of international articles have assessed both processes together. The novelty of this study is the use of primary data, which is representative of national production, and the combined assessment of broiler chicken meat and egg production. In addition, the results are presented from the perspective of 100 g of edible protein to aid when assessing the impacts of dietary guidelines in the future. Results from this study will add insight into an important food system that has grown substantially in the last thirty years and has become a staple in the Icelandic diet.

## Materials and methods

### Data collection

The data used was collected between September 2023 and June 2024 from interviews with local poultry and feed companies, personal communications, reports, and literature. Icelandic regulations (Regulation on Green Accounting, nr 851/2002) require that all companies, depending on the type of operation and size, that may cause pollution turn in Green Accounting reports. For poultry, these requirements refer to broiler chicken and laying hen farms with over 40,000 birds per rearing period (Regulation on Green Accounting, nr 851/2002). There are twenty-three farms that raise broiler chickens and ten farms that raise laying hens for eggs (MAST, 2025). Most of the poultry farms are small-scale raising flocks <40.000, therefore, they do not have to turn in green accounting reports. The reports used come from two of the biggest farming companies in the country, where the flocks range from 40.000 to 100.000 per rearing period, depending on the size of the farm. Four reports were available for broiler chicken farms (Environmental Agency of Iceland, 2022a,b,c,d), and two for laying hen farms (Environmental Agency of Iceland, 2022e, f) which represent approximately 60-70 % of the national production. An average of all the information gathered was calculated to form a more general picture of the production in Iceland for the year 2022.

Due to Iceland's small population, various important markets, such as broiler chicken meat and egg production, are each controlled by a few companies. Consequently, some of the data used for this article is sensitive and cannot be fully disclosed to secure normal ongoing competition between producers (Icelandic Competition Authority, 2024).

### Goal and scope definition

Two separate attributional LCAs were conducted to evaluate the environmental impacts of producing broiler chicken meat and eggs in Iceland according to the ISO 14040 and 14044 standards (ISO 14040, 2006; ISO 14044, 2006). The functional units for each LCA study (FU) were:

- 1) 1 kg of broiler chicken meat at carcass weight (CW), farmed in a conventional floor housing system in Iceland in 2022
- 2) 1 kg of eggs from laying hens in a barn housing system in Iceland in 2022

### System boundaries and allocation

The system boundary for broiler chicken meat production was cradle-to-slaughterhouse gate. This included the following production stages: parent birds (from 20 weeks), broiler chicken (for 35 days), feed production, manure management, and the slaughterhouse. The boundaries stop after slaughtering and before the broiler chicken meat is packaged and sent to retailers. This was done to be comparable with other literature findings in this field and due to the lack of data for the life cycle stages left out.

**Fig. 1:**
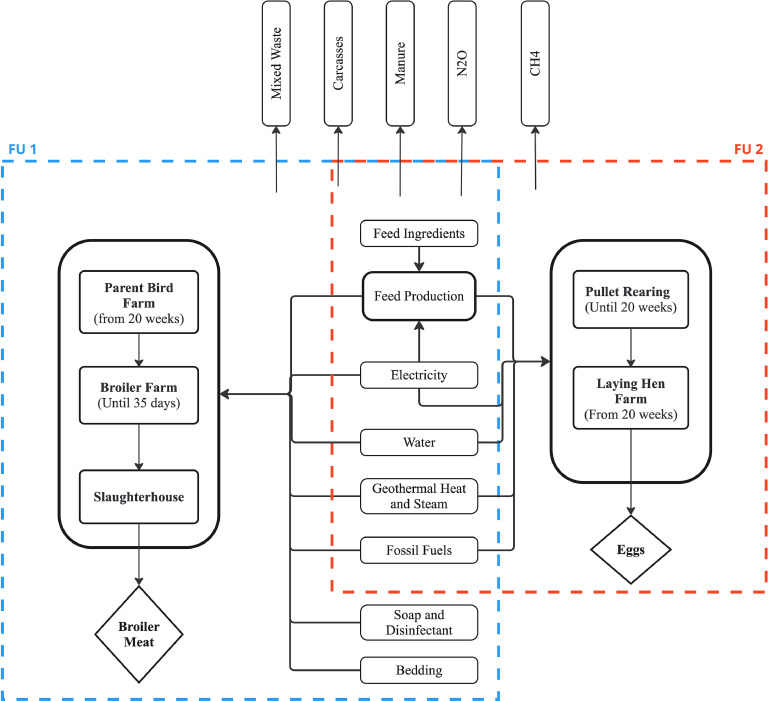
System boundaries for FU1 are marked with a blue dashed line and include all inputs and outputs for broiler chicken meat production. FU2 has red dashed lines and includes all inputs and outputs for egg production.

The system boundary for egg production was cradle-to-farm gate. The following stages were included: pullet rearing (before 20 weeks), laying hens (after 20 weeks), feed production, and manure management. Details of the system boundaries for broiler chicken meat and egg production are presented in Fig. 1. Broilers and laying hens have largely the same inputs in the system boundaries but in different quantities. Soap, disinfectant, and bedding were not included for the laying hen due to a lack of information. Mass allocation was used throughout the systems where multifunctionality was encountered.

### Life cycle inventory

***Feed.*** All feed recipes used were provided by two Icelandic feed companies. These companies import ingredients from abroad and mix the feed in Iceland. The proportions in the feed cannot be disclosed for confidentiality reasons, however, they were included in the LCA analyses.

Feed production for broiler chicken meat was accounted for in two separate phases: feed for parent birds and feed for broilers. The parent birds were assumed to be given two different types of feed depending on their age ranges, while broilers had three feed recipes (starter, grower, and finisher) for different stages of their life cycle.

In the case of laying hen, four feed recipes were provided. Two were used for the pullet rearing phase and two for the laying hen phase. The average amount of ingredients from two recipes for each phase was used for the pullet rearing and two for the laying hen stage.

***Farms.*** Broiler chickens are kept in a conventional floor housing system in Iceland. Data for this section was provided by leading Icelandic broiler company and supplemented with information from four green accounting reports (Table 1) (Environmental Agency of Iceland, 2022a,b,c,d).

**Table 1:** Inventory for broiler chicken meat production system according to the functional unit.

FU1- Broiler chicken	Unit	Broiler Chicken	Data Source	Parent birds	Data Source
Inputs					
Broiler	kg	1.00.*E*+00	Primary	Z	Z
Bird	birds	Z	Z	7.20.E-03	Primary
Feed	kg	1.98.*E*+00	Primary	2.51.E-01	Primary
Electricity	kWh	2.08.E-01	Primary	1.15.E-01	Primary
Oil	kg	6.15.E-03	Primary	1.21.E-02	Primary
Geothermal heat and steam	m^3^	1.04.E-01	Primary	1.60.E-02	Primary
Cold water	m^3^	3.88.E-03	Primary	1.18.E-03	Primary
Carcass	kg	1.12.E-02	Primary	3.89.E-02	Primary
Soap	kg	1.09.E-03	Primary	1.34.E-03	Primary
Disinfectant	kg	5.71.E-04	Primary	8.93.E-04	Primary
Bedding	kg	3.54.E-02	Primary	6.22.E-03	Primary
Outputs					
Mixed waste	kg	1.03.E-02	Primary	1.18.E-03	Primary
Solid waste	kg	8.27.E-03	Primary	4.92.E-04	Primary
Manure	kg	7.12.E-01	Primary	1.93.E-01	Primary
Slaughterhouse	Y	Y	Primary	Z	Z
Methane	kg CH_4_	1.28.E-03	FAO	1.71.E-04	FAO
Direct emissions	kg N_2_O	2.76.E-05	FAO	3.64.E-06	FAO
Indirect Emissions	kg N_2_O	1.11.E-04	IPCC	1.46.E-05	IPCC
Available for Soil	kg N_2_O	1.65.E-04	IPCC	2.17.E-05	IPCC

*Z*= does not apply, *Y*= confidential.

Data about the farm inventory for laying hens came from two Green Accounting reports, which account for two of the biggest egg production companies in the country (Table 2) (Environmental Agency of Iceland, 2022e,f). Most egg-laying farms use single- or multi-tier barn housing systems (Hansen, 2021). Information from the pullet rearing phase came from an Icelandic Carbon footprint report (Gíslason, S., Reykjavík, Personal communication).

**Table 2:** Inventory for the egg production system according to the functional unit.

FU2- Eggs	Unit	Pullets	Data Source	Laying hen	Data Source
Inputs					
Bird	Birds	Y	Secondary	Z	Z
Eggs	kg	Z	Z	1.00.*E*+00	Primary
Feed	kg	Y	Secondary	2.50.*E*+00	Primary
Electricity	kWh	Y	Secondary	2.81.E-01	Primary
Oil	kg	X	X	3.68.E-03	Primary
Geothermal heat and steam	m^3^	X	X	2.70.E-02	Primary
Cold water	m^3^	X	X	4.93.E-03	Primary
Outputs					
Carcass	kg	X	X	6.44.E-02	Primary
Mixed waste	kg	X	X	1.03.E-02	Primary
Manure	kg	Y	Secondary	6.18.E-01	Primary
Methane	kg CH_4_	1.90.E-04	FAO	2.39.E-03	FAO
Direct emissions	kg N_2_O	5.39.E-06	FAO	8.19.E-05	FAO
Indirect Emissions	kg N_2_O	2.16.E-05	IPCC	3.29.E-04	IPCC
Available for Soil	kg N_2_O	3.21.E-05	IPCC	4.90.E-04	IPCC

*X*= No information, *Y*= the amount is confidential, *Z* = does not apply.

***Slaughterhouse.*** The broiler chickens were sent to the slaughterhouse when they reached a marketable weight of 2-2.5 kg live weight within 32-35 days (Gíslason and Hallsdóttir, 2021). The laying period of parent birds and laying hens is assumed to be between 60 and 70 weeks. Most of the parent birds of broiler chickens and spent laying hens are disposed of and not sold for meat in Iceland and are thus not accounted for in the slaughterhouse stage. Carcass weight does not include viscera, feathers, head, and feet.

### Additional emission estimation

Manure naturally accumulates in animal farming, which can pose a serious threat to the environment as it can pollute the air and contaminate water and soil if not properly managed (Gržinić et al., 2023). In some countries, the manure generated exceeds the agricultural land available therefore correct storage and handling are important ways to prevent additional contamination or pollution (Gržinić et al., 2023). Additional calculations were done for the estimated methane (CH_4_) and nitrous oxide (N_2_O) emissions that are generated from the manure for both poultry systems in Iceland following the LEAP/ FAO (2016) and IPCC (2019) guidelines (formulas used are provided as supplementary material). The manure was assumed to be a waste product with no economic value.

### Life cycle impact assessment and data handling

The life cycle impact assessment (LCIA) method used was the CML-IA Baseline (3.08- EU25), which has eleven environmental impact categories (Guinée et al., 2002). The following impact categories were of special focus: Global Warming Potential (GWP), Abiotic Depletion Potential (fossil fuel), Acidification Potential, and Eutrophication Potential, both because of their relevance to feed production and manure management and their frequent analysis in literature (Costantini et al., 2021). Microsoft Excel was used for data extraction and handling, SimaPro 9.4 was used for the LCA analyses, using the Ecoinvent 3.8- cut-off unit library for background processes (PRé Consultants, 2016; Simapro, 2024).

### Sensitivity analysis

The respective baseline scenarios for broiler chicken meat and egg production were 1) the production of broiler chicken in a conventional floor housing system and 2) laying hens in a barn housing system for egg production. Two additional scenarios were considered to assess the possible environmental impact of allocating based on protein content: 3) producing 100g of edible protein from broiler chicken meat, and 4) producing 100g of edible protein from eggs. For scenario 3, the total edible protein in a whole chicken and each chicken cut was calculated using available data on the nutritional value of Icelandic poultry meat and the average amount of meat in chicken wings (Reykdal and Hilmarsson, 2020; Nakano and Ozimek 2015). Those findings were adapted to fit the estimated slaughter weight used in this study (broiler chicken at 1.68 kg (CW) estimated to contain 195 g of edible protein). For scenario 4, the weight of an average egg in Iceland was assumed to be 63 g, of which 13 % of the weight is the shell and 87 % is the edible part (ÍSGEM, 2003). Therefore, approximately 13.9 eggs (877 g) contain 100 g of edible protein

An additional sensitivity analysis was conducted for soybean meal, assuming it was sourced from Brazil (Ecoinvent process *Market for Soybean Meal, Brazil)* or from an average world market (Ecoinvent process, *Market for Soybean Meal, Rest of the World-* RoW).

## Results and discussion

The total values for each environmental impact category to produce 1 kg of broiler chicken (CW) at slaughterhouse gate, and 1 kg of eggs at farm gate are displayed in Table 3. The table shows the results for all eleven impact categories, and the following chapters will discuss the results for the four selected impact categories: Global Warming Potential, Abiotic Depletion Potential (fossil fuel), Acidification Potential, and Eutrophication Potential.

**Table 3:** The LCIA results for the estimated environmental impact of 1 kg of broiler chicken meat at carcass weight (CW), 1 kg of eggs, and 100 g of edible protein from both poultry sources.

Impact Categories	Units	1 kg Broiler (CW)	100g. Edible Protein (Meat)	1 kg Eggs	100g. Edible Protein (Eggs)
**Global warming**	**kg CO_2_ eq**	**2.82.*E*+00**	**2.41.*E*+00**	**2.38.*E*+00**	**2.05.*E*+00**
**Abiotic depletion (fossil fuels)**	**MJ**	**1.16.*E*+01**	**9.92.*E*+00**	**1.30.*E*+01**	**1.12.*E*+01**
**Acidification**	**kg SO_2_ eq**	**1.40.E-02**	**1.20.E-02**	**1.45.E-02**	**1.25.E-02**
**Eutrophication**	**kg PO_4_ eq**	**1.25.E-02**	**1.07.E-02**	**1.08.E-02**	**9.29.E-03**
Abiotic depletion	kg Sb eq	1.51.E-05	1.29.E-05	1.52.E-05	1.31.E-05
Ozone layer depletion (ODP)	kg CFC-11 eq	1.28.E-07	1.09.E-07	2.93.E-07	2.52.E-07
Human toxicity	kg 1.4-DB eq	9.96.E-01	8.52.E-01	9.84.E-01	8.46.E-01
Fresh water aquatic ecotox.	kg 1.4-DB eq	1.01.*E*+00	8.64.E-01	1.11.*E*+00	9.55.E-01
Marine aquatic ecotoxicity	kg 1.4-DB eq	1.18.*E*+03	1.01.*E*+03	1.27.*E*+03	1.09.*E*+03
Terrestrial ecotoxicity	kg 1.4-DB eq	6.56.E-02	5.61.E-02	2.34.E-02	2.01.E-02
Photochemical oxidation	kg C_2_H_4_ eq	1.32.E-03	1.13.E-03	8.22.E-04	7.07.E-04

**Fig. 2:**
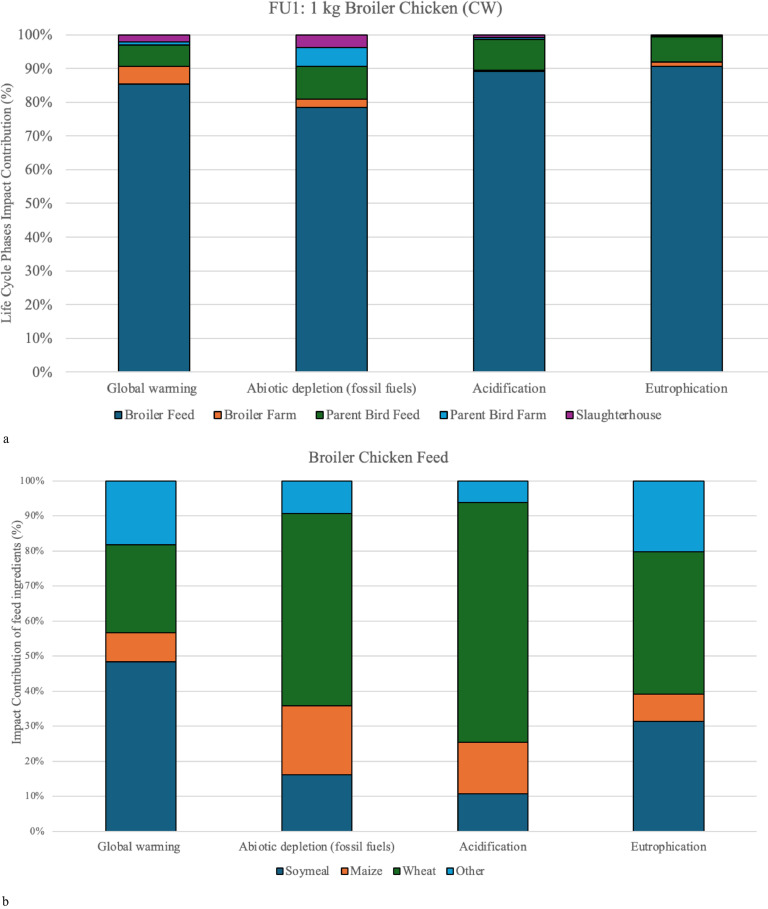
a) Impact contribution of life cycle phases for FU1, 1 kg broiler chicken at CW, and b) impact contribution of feed ingredients in the broiler feed.

**Fig. 3:**
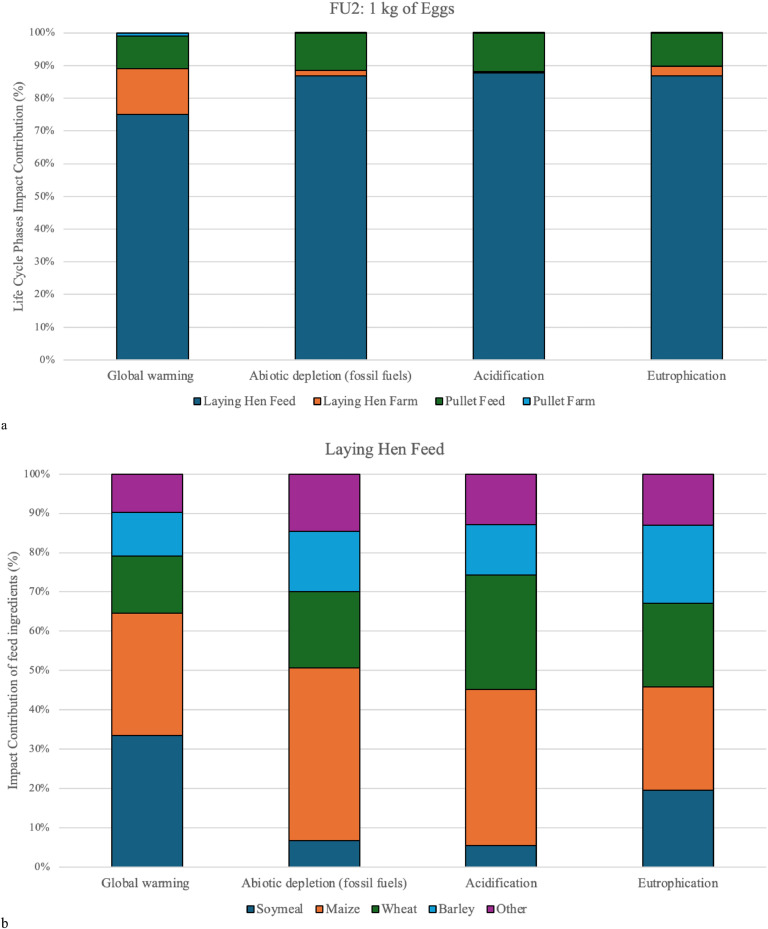
a) Impact contribution of life cycle phases for FU2, 1 kg egg production, and b) impact contribution of feed ingredients in laying hen feed.

The impact contribution for each life cycle phase for these four selected impact categories are shown in Fig. 2a and 3a. Feed production is the main environmental hotspot across the assessed impact categories for both food systems, Fig. 2b and 3b show the impact of the feed ingredients.

The broiler feed alone ranges between 80 % and 90 % of the total impact in the respective impact categories for broiler chicken meat production (FU1) (Fig. 2a). Similar results can be seen for egg production (FU2), where the feed for the laying hen phase generates between 75 % and 91 % of the total impact in respective categories (Fig. 3a). These findings align with general results in the literature (González-García et al., 2014; Leinonen et al., 2012a, b; Cesari et al., 2017; Kalhor et al., 2016).

The environmental impact derived from the feed is primarily due to a handful of ingredients: wheat (9-63 %), soybean meal (10-71 %), and maize (9-25 %) for the broiler system (Fig. 2b), and wheat (12-29 %), soybean meal (12-64 %), barley (10-41 %), and maize (16-57 %) for eggs (Fig. 3b). Collectively, they contribute between 61 % and 90 % of the total impact from feed for both poultry systems depending on the impact category.

The impact generated at the poultry farms for both production systems come from emissions from manure, diesel oil used for the farm machinery, and waste.

Soap, disinfectant, and bedding had a low impact contribution to the broiler system (less than 1 % for all impact categories). These inputs were not accounted for in the egg production LCA model due to lack of information, however considering the results it had on the broiler system, it was expected to have minimal impact.

Overall, the impacts of the farm life cycle stage are low compared to the other stages, which could be attributed to the utilization of renewable energy and geothermal heating on a country level.

### Additional emission estimation

This chapter shows the impact estimations from the additional emissions (N_2_O and CH_4_) generated from the manure and its management.

The combined effects from N_2_O and CH_4_ emissions towards the GWP for FU1 were 0.13 kg CO_2_ eq/kg CW or 5 % of the total GWP impact, while for FU2, the impact was 0.33 kg CO_2_ eq/ kg egg or 14 % of total GWP impact. In the broiler chicken production, other impact categories were not affected by the manure emissions except for Eutrophication Potential and Photochemical Oxidation, which had an impact contribution below 1 % each. In the egg production system, N_2_O and CH_4_ emissions had an impact contribution of 2.4 % Eutrophication Potential and 2.2 % on Photochemical Oxidation. Moreover, based on the IPCC factors used for poultry manure with litter, no leaching of N_2_O emissions was estimated from the management of manure (Table 1, Table 2, Table 4).

**Table 4:** Results for the calculations of manure management according to the functional unit.

Method & Emissions	Unit	Equation	1 kg Broiler	1 kg Eggs
FAO, 2016				
Methane Emissions	Kg CH_4_	Eq 6	1.44E-03	2.58E-03
Direct Nitrous Oxide Emissions	Kg N_2_O	Eq 7	3.13E-05	8.73E-05
IPCC, 2019				
Manure Management Indirect	Kg N_2_O	Eq 10.28	1.25E-04	3.50E-04
Manure Management Leaching	Kg N_2_O	Eq 10.29	0.00E+00	0.00E+00
Direct N_2_O emissions from Managed soils	Kg N_2_O	Eq. 11.1	1.86E-04	5.22E-04

### Selected impact categories

To compare the results of this study with findings in the literature, studies with similar system boundaries and the same housing systems were considered (Table 5), as free-range and organic housing systems are generally not found in Iceland and are, therefore less compatible. The rearing period of broiler chickens can vary between countries, the studies considered have a range between 34 and 50 days, this is generally due to the breed of broiler chickens farmed and the nutrition composition of the feed.

**Table 5:** Results, methods, and system boundaries used in literature and this study.

Authors	Functional Unit	LCIA Method	System boundary	Housing System	GWP	ADPF	AP	EP
					kg CO_2_eq	MJ eq	kg SO_2_eq	kg PO_4_ eq
Cesari et al.	1 kg CW	CML	C-T-S^a^	CVN^b^	5.52	20.3^h^	0.028	0.018
García et al.	1.2 kg CW	CML	C-T-S	CVN	3.00	22.2^h^	0.053	0.025
Kalhor et al.	1 ton CW	CML	C-T-S	CVN-Summer	2.93	x	0.042	0.015
	1 ton CW	CML	C-T-S	CVN -Winter	5.35	x	0.062	0.019
Leinonen et al. (a)	1 ton CW	CML	C-T-S	CVN	4.41	25.4	0.047	0.020
da Silva Lima et al.	1 kg LW	CML	C-T-F^c^	CVN	2.70	x	0.040	0.026
Mostert et al.	1 kg LW	Feed Print	C-T-F	CVN	3.65	x	x	x
Ogino et al.	1 kg LW	CML	C-T-F	CVN	1.86	18.8	0.053	0.018
Skunca et al.	1 kg of Consumed Broiler Meat	IMPACT	C-T-G^d^	CVN	3.62	48.7	0.081	x
Usva et al.	1 kg CW	IPCC, AWARE	C-T-S	CVN	2.37	x	x	x
Gíslason et al.	1 kg CW	Carbon Footprint study	C-T-S	CVN	2.25	x	x	x
**This study**	**1kg CW**	**CML**	**C-T-S**	**CVN**	**2.82**	**11.6**	**0.014**	**0.012**
Turner et al.	1 tonn Eggs	CML	C-T-F	Barn	2.40	x	0.081	0.028
Guillaume et al.	1 kg Shelled Eggs	Environmental footprint 3.0	C-T-F	Barn	3.45	14.95	0.12^e^	0.48^f^
Dekker et al.	1 kg Eggs	IPCC 2006	C-T-F	Barn single-tier	2.68	23.2	0.063	x
	1 kg Eggs	IPCC 2006	C-T-F	Barn multi-tier	2.66	22.5	0.040	x
Leinonen et al. (b)	1 tonn eggs	IPCC 2006	C-T-F	Barn	3.45	22.2	0.059	0.020
Pelletier	1 tonn of packaged eggs	CML	C-T-R^g^	Barn	2.40	11.8	0.080	0.027
**This study**	**1kg Eggs**	**CML**	**C-T-F**	**Barn**	**2.38**	**13.0**	**0.015**	**0.011**

a C-T-S: Cradle to Slaughterhouse, ^b^ CVN: Conventional, ^c^C-T-F: Cradle to Farm gate, ^d^C-T-G: Cradle to Grave, ^e^Results are in Molc *H*+ eq, ^f^Results are in g P eq, ^g^C-T-R: Cradle to retail, ^h^Cumulative energy demand, x:not analyzed.

Additionally, it is important to keep in mind when comparing results from different LCIA methods and allocation choices can affect the results.

***Global Warming Potential (GWP).*** GWP considers the combined greenhouse gas emissions that are released from processes into the atmosphere during the life cycle of a product (Leinonen and Kyriazakis, 2016). In the production of broiler chicken meat, the total GWP was 2.82 kg CO_2_ eq/kg CW, of which 91 % came from total feed production (parent bird feed and broiler chicken feed) (Fig. 2a), 5 % from manure management, and the remaining 4 % came from bird farming stages.

The Finnish broiler chicken production seems to be the most comparable to the Icelandic production life cycle when considering the input and outputs used in their study (Usva et al., 2023). The results presented by Usva et al. (2023) showed the GWP to be slightly lower, 2.37 kg CO_2_ eq/kg CW. Finland produces domestically high shares of the feed ingredients (73 %), which could play a part in lower emissions, as seen with the feed contribution, which is only 79 % of the total impact. Other studies generally reported higher emissions ranging between 3.00 to 5.52 kg CO_2_ eq/kg CW (Table 5) (González-García et al., 2014; Cesari et al., 2017; Skunca et al., 2018; Mostert et al., 2022). One carbon footprint report is available for Icelandic broiler chicken meat production, where results estimate a lower GWP impact, or 2.25 kg CO_2_ eq/kg CW (Gíslason and Hallsdóttir, 2021). Although any direct comparison between GHG emission protocol method and LCA is not optimal, both studies broadly identified feed production as having the most significant impact (Gíslason and Hallsdóttir, 2021).

For egg production, the total GWP for 1 kg of eggs was 2.38 kg CO_2_ eq, of which total feed (pullet feed and laying hen feed) production was 85 % of the impact (Fig. 3a) and 14 % due to manure management which is connected to the higher feed conversion rate when compared to the broiler chicken meat system. Results from an unpublished carbon footprint report on Icelandic egg production estimated 2.02 kg CO_2_ eq/kg eggs (Gíslason, S., Reykjavík, Personal communication). Overall, the results seem to be similar or lower than many findings in the literature, where the results vary from 2.40 to 3.45 kg CO_2_ eq/kg eggs (Table 5) (Turner et al., 2022; Pelletier, 2017; Leinonen et al., 2012b).

***Abiotic Depletion Potential (fossil fuel, ADPF).*** ADPF refers to the use of energy carriers that are from non-renewable sources such as natural gas, coal, and oil (Van Oers and Guinée, 2016).

The results for the broiler chicken were 12 MJ/kg CW (Table 3), which was mainly due to the use of crude oil and hard coal during the feed production (88 %) (Fig. 2a). However, the combined farming stages for both broilers and parent birds had an 8 % impact contribution on this category which was primarily due to the use of diesel oil for farm equipment. The availability of renewable energy in Iceland might have been the reason for the low values, as many countries rely on fossil fuels for heating and generating electricity for energy-intensive stages such as farms and slaughterhouses, as seen in Leinonen et al. (2012a). They found that the primary energy used for 1 kg broiler chicken farmed in a standard housing system in the UK was 25 MJ eq (Leinonen et al., 2012a). The ADPF impact was largely connected to feed production (64 %), heating from gas and oil (12-25 %), and electricity (11 %), highlighting the additional impact that can come from non-renewable energy sources (Leinonen et al., 2012a).

The ADPF results for egg production were 13 MJ/kg eggs, which is slightly higher than broiler chicken meat impact results for this category (Table 3). These results are almost exclusively connected to feed production for both the pullets and laying hen stages (98 %). The egg production had a higher feed conversion rate than the broiler chicken meat system (2.5 kg feed/1 kg eggs versus 1.9 kg feed/1 kg CW), resulting in a slightly higher impact in this category. However, Pelletier (2017) reported 11.8 MJ/ kg eggs, which is similar to the findings of this study, while Dekker et al. (2011) and Leinonen et al. (2012b) had the higher impact of 22-23 MJ/ kg eggs (Table 5).

***Acidification Potential (AP).*** AP primarily indicates the potential for a reduction of pH in the soil and water, the major sources of acidification from poultry farming come from the ammonium (NH_3_) emissions generated from the manure and sulfur dioxide (SO_2_) from the combustion of fossil fuel (Leinonen and Kyriazakis, 2016). Ventilation and litter control are essential to dry out the manure during the barn stage, slowing the decomposition process and reducing NH_3_ emissions (Swelum et al., 2021).

The results for the acidification potential for *both poultry systems* were 0.014 kg SO_2_ eq/kg CW and 0.015 kg SO_2_ eq/kg eggs (Table 3). The barns in Iceland have good control of heating and ventilation, leaving the manure dry, which is generally removed after every production cycle. This study did not include additional estimations for ammonia emissions, which could explain the low outcome. Therefore the AP results are exclusively related to the feed production (98-99 %) linked to fertilizers used for wheat, maize, and soybean production (Fig. 2b). These AP findings are lower than what is found in literature, ranging between 0.028-0.062 kg SO_2_ eq/kg CW and 0.040-0.081 kg SO_2_ eq/kg egg (Table 5) (Cesari et al. 2017; Ogino et al., 2021; Kalhor et al., 2016; Dekker et al., 2011; Turner et al., 2022). It has been suggested that the wide range of results can be explained by the different methods used to estimate the emissions (da Silva Lima et al., 2019; Cesari et al., 2017).

***Eutrophication Potential (EP).*** EP is a measure of potential excessive nutrient supplies in water systems that lead to algae blooms and ecological imbalance (Chislock et al., 2013). Usually, these nutrients reach water sources through leaching, runoff, or atmospheric deposition (Leinonen and Kyriazakis, 2016). Examples of these processes include nitrate and phosphate leaching into water systems or ammonia (NH_3_) emissions into the atmosphere (Leinonen and Kyriazakis, 2016).

According to the calculations shown in Table 4, no leaching from the manure management is estimated. Therefore, the EP results were exclusively (97-98 %) explained by the feed production due to fertilizers used, for both broiler and egg production systems. The EP results for broiler chicken meat were 0.013 kg PO_4_ eq/kg CW and 0.011 kg PO_4_ eq/kg egg for egg production (Table 3). These slight differences between the EP results for laying hens and broiler chickens were due to different choices of feed ingredients (Fig. 2b and Fig. 3b).

The EP results were similar to Kalhor's (2016) findings of 0.015-0.019 kg PO_4_ eq/kg CW, whereas González-García et al. (2014) had higher results of 0.025 kg PO_4_ eq/kg CW. While for egg production, the results in the literature ranged between 0.020 and 0.027 kg PO_4_ eq/kg egg (Table 5) (Leinonen et al., 2012b; Turner et al., 2022).

### Sensitivity analysis

The specific impacts of feed production can vary, both due to management decisions and differences in natural conditions between countries. Therefore, a sensitivity analysis was conducted to see if and how the selection of different production processes within the selected database (Ecoinvent) could affect the result of feed production. Results from this study showed that soybean meal is one of the ingredients with a higher contribution to selected impact categories in broiler chicken meat production. Therefore, the process used for this study, *Market for Soybean Meal, Brazil (baseline scenario*) was compared to the process *Market for Soybean Meal, Rest of World (RoW)* to see how sensitive this system was to a change in the process selection. The percentage change (Table 6) of selecting the RoW soybean meal market process over the Brazil market process was calculated for each impact category for broiler chicken meat production. A positive percentage change indicates a higher LCIA result in the alternate scenario compared to the baseline, whereas a negative percentage change indicates a lower environmental impact for the alternative scenario.

**Table 6:** Sensitivity analysis results from selecting two different geographies available in Ecoinvent for the market process of soybean meal. LCIA results displayed are for total broiler feed production.

Impact Category	Brazil (Baseline)	Rest of World	Percentage change
Global warming (GWP100a)	2.4.*E*+00	2.9.*E*+00	19 %
Abiotic depletion (fossil fuels)	9.1.*E*+00	9.9.*E*+00	8 %
Acidification	1.3.E-02	1.2.E-02	−1 %
Eutrophication	1.1.E-02	9.8.E-03	−13 %
Abiotic depletion	1.3.E-05	1.1.E-05	−12 %
Ozone layer depletion (ODP)	8.7.E-08	9.7.E-08	12 %
Human toxicity	8.5.E-01	7.3.E-01	−13 %
Fresh water aquatic ecotox.	8.0.E-01	1.0.*E*+00	27 %
Marine aquatic ecotoxicity	8.5.*E*+02	8.4.*E*+02	−2 %
Terrestrial ecotoxicity	6.3.E-02	2.0.E-01	220 %
Photochemical oxidation	1.2.E-03	8.5.E-04	−30 %

The comparison in Table 6 indicates that replacing the soybean meal from *Brazil* market process with *RoW*, reduces the potential environmental impact of six categories but increases the impacts in the remaining five categories. If RoW market soybean meal process is used instead of the Brazil market process, the GWP of feed production required to produce 1 kg broiler chicken at CW would increase by 19 %. Feed represents the most significant environmental impact in the life cycle of broiler chicken meat, switching between these processes would therefore affect the total results of GWP, increasing them from 2.82 to 3.28 kg CO_2_ eq/kg CW. When considering the other three selected impact categories, these results caused either a minor or moderate change in impact. *Market for Soybean Meal, Brazil* was selected for this study to be in line with the information gathered for feed ingredients used in Iceland. These sensitivity results show that the geographical definition of selected process can affect the LCIA results.

### Edible protein from chickens

#### 100 g of edible protein from broiler chicken meat

**Fig. 4:**
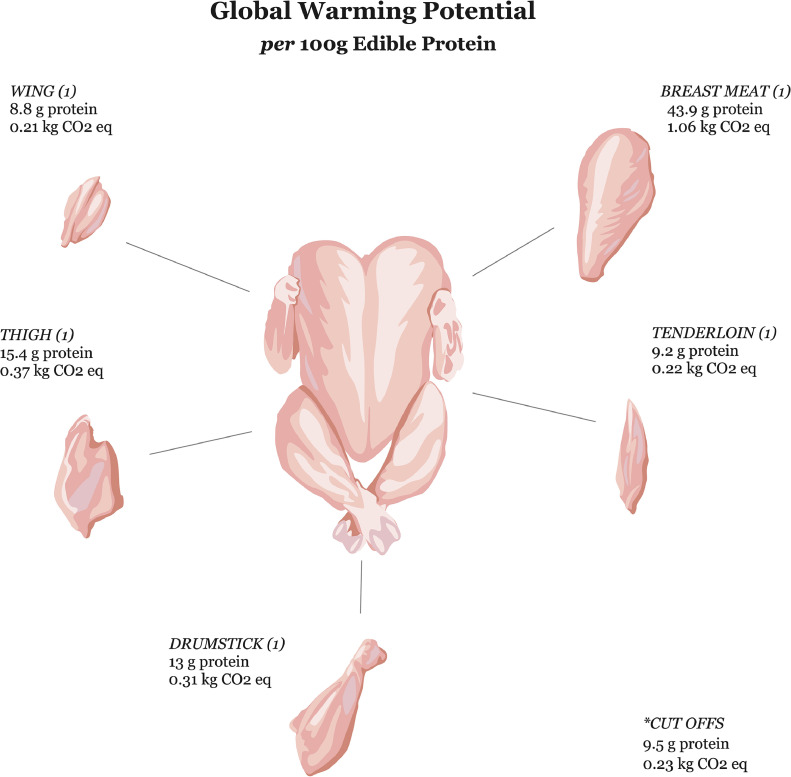
The estimated global warming potential of different chicken cuts from the perspective of 100 g of edible protein. Each cut shows the amount of protein and estimated impact per one piece.

The environmental impacts were considered from the perspective of 100 g of edible protein, which resulted in 2.41 kg CO_2_ eq for GWP (Table 3). Because chicken is commonly sold by cuts, it was also determined that each cut should be considered separately. The estimated amount of GWP was then allocated to different chicken cuts based on their edible protein content (Table 7 and Fig. 4).

**Table 7:** Edible protein content in each chicken cut and its estimated distribution of global warming potential.

Chicken Cut	Weight (meat only)	Protein	GWP
	g	g	kg CO_2_ eq
Breast meat	198	43.9	1.06
Tenderloin	40	9.2	0.22
Thigh	92	15.4	0.37
Drumstick	70	13.0	0.31
Wing	47	8.8	0.21
Cut offs	48	9.5	0.23
Ribcage	x	x	Zero allocated
Skin and Bones	x	x	Zero allocated
Total	495	100	2.41

The carcass was split into six specific cuts: one breast meat, one tenderloin, one thigh, one drumstick, one wing, and cut-offs (without skin and bones). These parts represent approximately 60 % of the CW, with 22 % of the remaining weight coming from the ribcage and 18 % from skin and bones collectively. The protein-based impact allocation was conducted to all cuts, except for skin and bones due to their lack of edible protein.

***100g of edible protein from eggs.*** The results showed that 100 g of edible protein from eggs had a GWP of 2.05 kg CO_2_ eq (Table 3). 100 g of protein derived from eggs has 16 % lower carbon emissions than those from broiler chicken meat; however, these comparisons are only from an environmental impact perspective, not a nutritional comparison.

#### Limitations of this study

The study had several limitations, primarily due to the lack of data for certain aspects. These excluded elements are soap, disinfectant, and bedding inputs for the life cycle of laying hens. Additionally, the hatchery stage was not considered, and emissions from transportation between life cycle stages of both systems. Packaging was not included, as it falls outside the system boundaries of this study. Including these additional stages and inputs would enhance the accuracy of the overall results; however, previous studies (Guillaume et al., 2022; Turner et al., 2022) have shown that their impact on the life cycle is minimal. Other processes, such as medicines, machinery, buildings, and maintenance, were also excluded, following standard practice in the literature (Cesari et al., 2017).

#### Recommendations for future studies

Spent hens are generally regarded as low-quality meat, with only a small percentage sold for human consumption (Costantini et al., 2020). While the rest are disposed of in different ways such as landfills, utilized in animal feed, or incinerated (Pelletier, 2017). This study assumed that they were sent to landfills. Further research is needed to explore ways to utilize spent hens more effectively.

The Life Cycle Impact Assessment (LCIA) used in this study does not account for plastic pollution or microplastics and their environmental impacts. Future studies should incorporate methodologies that assess microplastics, given their potential effects on the environment and animal health (Lu et al., 2024). Additionally, more research is required to estimate the potential benefits of applying manure to various types of soil or land in Iceland as a substitute for synthetic nitrogen fertilizer.

## Conclusion

With the growing concern about emission-intensive food production, future nutritional guidelines should consider the environmental impact associated with the life cycle of food systems to lower the consumption of emission-intensive foods. For this reason, this study estimated the environmental impact of a major food systems in Iceland, broiler chicken meat and egg production. These results are expected to represent approximately 60-70 % of the national production of both systems.

The overall results showed a lower environmental impact in all four selected impact categories when compared with international findings. The global warming potential (GWP) was 2.82 kg CO_2_ eq/kg CW and 2.38 kg CO_2_ eq/kg eggs. The GWP difference between broiler chicken meat and egg production was due to the feed conversion ratio and differences in feed recipes. While feed production was the major environmental hotspot in both food systems, specific ingredient contributions changed among product systems. For example, wheat and soybean were the major environmental contributors to broiler chicken meat production, whereas maize was the major contributor to egg production.

Iceland's poultry sector relies on imports for nearly all feed ingredients. Therefore, considering the source of ingredients and the proportion of selected ingredients used in feed recipes can further reduce some environmental impacts.

To further integrate the results with dietary information, the impact was expressed for 100 g of edible protein for both broiler chicken meat and eggs. This was done to provide additional information and to support the development of future dietary guidelines that will also consider environmental impact.

## Declaration of competing interest

The authors declare the following financial interests/personal relationships which may be considered as potential competing interests:

Olafur Ogmundarson reports financial support was provided by ERA-Net Cofund scheme. Olafur Ogmundarson reports financial support was provided by The Icelandic Centre for Research. If there are other authors, they declare that they have no known competing financial interests or personal relationships that could have appeared to influence the work reported in this paper.

## Appendix Supplementary materials

Image, application 1

## References

[bib0001] Alifuglahald. (2024). MAST: https://www.mast.is/is/baendur/alifuglaraekt/alifuglahald.

[bib0002] Blomhoff R., Andersen R., Arnesen E.K., Christensen J.J., Eneroth H., Erkkola M., Gudanaviciene I., Halldórsson Þ.I., Höyer-Lund A., Lemming E.W. (2023). Nordic Nutrition Recommendations 2023: Integrating Environmental Aspects.

[bib0003] Cesari V., Zucali M., Sandrucci A., Tamburini A., Bava L., Toschi I. (2017). Environmental impact assessment of an Italian vertically integrated broiler system through a Life cycle approach. J. Clean. Prod..

[bib0004] Cheng M., McCarl B., Fei C. (2022). Climate change and livestock production: a literature review. Atmosphere (Basel).

[bib0005] Chislock M.F., Doster E., Zitomer R.A., Wilson A.E. (2013). Eutrophication: causes, consequences, and controls in aquatic ecosystems. Nat. Educ. Knowl..

[bib0006] Conijn J.G., Bindraban P.S., Schröder J.J., Jongschaap R.E.E. (2018). Can our global food system meet food demand within planetary boundaries?. Agric. Ecosyst. Environ..

[bib0047] Consultants P.R.é (2016). http://www.pre.nl.

[bib0007] Costantini M., Ferrante V., Guarino M., Bacenetti J. (2021). Environmental sustainability assessment of poultry productions through life cycle approaches: a critical review. Trends. Food Sci. Technol..

[bib0008] Costantini M., Lovarelli D., Orsi L., Ganzaroli A., Ferrante V., Febo P., Bacenetti J. (2020). Investigating on the environmental sustainability of animal products: the case of organic eggs. J. Clean. Prod..

[bib0009] da Silva Lima N.D., de Alencar Nääs I., Garcia R.G., de Moura D.J. (2019). Environmental impact of Brazilian broiler production process: evaluation using life cycle assessment. J. Clean. Prod..

[bib0010] Dekker S., De Boer I.J., Vermeij I., Aarnink A.J., Koerkamp P.G. (2011). Ecological and economic evaluation of Dutch egg production systems. Livest. Sci..

[bib0011] Environmental Agency of Iceland. (2022a). Green Accounting- Matfugl. https://ust.is/library/sida/atvinnulif/starfsleyfi-og-eftirlitsskyrslur/Grænt%20bókhald%20Hurðarbak_2022.pdf.

[bib0012] Environmental Agency of Iceland. (2022b). Green Accounting- Matfugl. https://ust.is/library/sida/atvinnulif/starfsleyfi-og-eftirlitsskyrslur/Grænt%20bókhald%20Melavellir%202022.pdf.

[bib0013] Environmental Agency of Iceland. (2022c). Green accounting- Reykjagarður. https://ust.is/library/sida/atvinnulif/starfsleyfi-og-eftirlitsskyrslur/Ásmundarstaðir%202022%20undirritað.pdf.

[bib0014] Environmental Agency of Iceland. (2022d). Green accounting- Reykjagarður. https://ust.is/library/sida/atvinnulif/starfsleyfi-og-eftirlitsskyrslur/Jarlsstaðir%202022%20undirritað.pdf.

[bib0015] Environmental Agency of Iceland. (2022e). Green accounting- Stjörnuegg. https://ust.is/atvinnulif/mengandi-starfsemi/starfsleyfi/thauleldi/stjornuegg-valla-kjalarnesi/.

[bib0016] Environmental Agency of Iceland. (2022f). Green accounting- Nesbúegg. https://ust.is/library/sida/atvinnulif/starfsleyfi-og-eftirlitsskyrslur/Grænt%20bókhald%20Nesbúeggja%202022_loka.pdf.

[bib0017] FAO (2016).

[bib0018] FAO (2022).

[bib0019] FAO (2023). Pathways towards Lower Emissions -A Global Assessment of the Greenhouse Gas Emissions and Mitigation Options from Livestock Agrifood Systems.

[bib0020] Gíslason S., Hallsdóttir B.S. (2021). Kolefnisfótspor Íslenskra Kjúklinga Og Leiðir Til Að Minnka Það.

[bib0021] González-García S., Gomez-Fernández Z., Dias A.C., Feijoo G., Moreira M.T., Arroja L. (2014). Life Cycle Assessment of broiler chicken production: a Portuguese case study. J. Clean. Prod..

[bib0022] Government of Iceland. (2023). Mælaborð Landbúnaðarins. https://www.stjornarradid.is/verkefni/atvinnuvegir/landbunadur/maelabordlandbunadarins.

[bib0023] Government of Iceland. (2024). Energy. https://www.government.is/topics/business-and-industry/energy/.

[bib0024] Gržinić G., Piotrowicz-Cieślak A., Klimkowicz-Pawlas A., Górny R.L., Ławniczek-Wałczyk A., Piechowicz L., Wolska L. (2023). Intensive poultry farming: a review of the impact on the environment and human health. Sci. Total Environ..

[bib0025] Guillaume A., Hubatová-Vacková A., Kočí V. (2022). Environmental impacts of egg production from a life cycle perspective. Agriculture.

[bib0027] Guinée J., Gorrée M., Heijungs R., Huppes G., Kleijn R., Koning A.d., Oers L.v., Wegener Sleeswijk A., Suh S., Udo de Haes H.A., Bruijn H.d., Duin R.v., Huijbregts M.A.J (2002).

[bib0028] Gunnarsdóttir S., Guðmannsdóttir R., Thorgeirsdóttir H., Torfadóttir J.E., Steingrimsdóttir L., Tryggvadóttir E.A. (2022). Könnun Á Mataræði Íslendinga 2019-2021 (What do Icelanders eat? Survey of the diet of Icelanders 2019-2021).

[bib0029] Hansen V. (2021). Allar Hænur Í Lausagöngu.

[bib0035] ÍSGEM (2003). https://matis.is/wp-content/uploads/2021/03/Naering_egg.pdf.

[bib0031] ISO 14040 (2006).

[bib0032] ISO 14044 (2006).

[bib0033] IPCC (2019). Chapter 10 Emissions from Livestock and Manure Management in: 2019 Refinement to the 2006 IPCC Guidelines for National Greenhouse Gas Inventories Volume 4: Agriculture.

[bib0030] Icelandic Competition Authority (2024). https://www.samkeppni.is/malefni/ologmaett-samrad/hagsmunasamtok-fyrirtaekja-og-samkeppnisreglur/.

[bib0034] IPCC (2022). Contribution of Working Group II to the Sixth Assessment Report of the Intergovernmental Panel on Climate Change.

[bib0037] Kalhor T., Rajabipour A., Akram A., Sharifi M. (2016). Environmental impact assessment of chicken meat production using life cycle assessment. Inform. Process. Agricult..

[bib0038] Leinonen I., Kyriazakis I. (2016). How can we improve the environmental sustainability of poultry production?. Proc. Nutrit. Soc..

[bib0039] Leinonen I., Williams A., Wiseman J., Guy J., Kyriazakis I. (2012). Predicting the environmental impacts of chicken systems in the United Kingdom through a life cycle assessment: broiler production systems. Poult. Sci..

[bib0040] Leinonen I., Williams A., Wiseman J., Guy J., Kyriazakis I. (2012). Predicting the environmental impacts of chicken systems in the United Kingdom through a life cycle assessment: egg production systems. Poult. Sci..

[bib0041] Lu H., Guo T., Zhang Y., Liu D., Hou L., Ma C., Xing M. (2024). Endoplasmic reticulum stress-induced NLRP3 inflammasome activation as a novel mechanism of polystyrene microplastics (PS-MPs)-induced pulmonary inflammation in chickens. J. Zhejiang Univ. Sci. B.

[bib0043] MAST (2025). Kortasjá.

[bib0036] Mostert P., Bos A., Van Harn J., De Jong I. (2022). The impact of changing toward higher welfare broiler production systems on greenhouse gas emissions: a Dutch case study using life cycle assessment. Poult. Sci..

[bib0042] Nakano T., Ozimek L. (2015). A method of production of boneless chicken wings (drumettes and winglets) by separation of periosteum from bone without cutting skin and muscles. Poult. Sci..

[bib0044] Ogino A., Oishi K., Setoguchi A., Osada T. (2021). Life cycle assessment of sustainable broiler production systems: effects of low-protein diet and litter incineration. Agriculture.

[bib0045] Olgeirsson F.G. (2003). Alifuglinn: saga alifuglaræktar Á Íslandi frá Landnámi Til okkar daga.

[bib0046] Pelletier N. (2017). Life cycle assessment of Canadian egg products, with differentiation by hen housing system type. J. Clean. Prod..

[bib0048] Regulation on Green Accounting nr 851. (2002). https://island.is/reglugerdir/nr/0851-2002.

[bib0049] Reykdal Ó., Hilmarsson Ó.Þ. (2020). Nýting og næringargildi íslensks alifuglakjöts.

[bib0050] Sala S., McLaren S.J., Notarnicola B., Saouter E., Sonesson U. (2017). In quest of reducing the environmental impacts of food production and consumption. J. Clean. Prod..

[bib0052] Skunca D., Tomasevic I., Nastasijevic I., Tomovic V., Djekic I. (2018). Life cycle assessment of the chicken meat chain. J. Clean. Prod..

[bib0053] Statistics Iceland (2024). https://px.hagstofa.is/pxis/pxweb/is/Atvinnuvegir/Atvinnuvegir__landbunadur__landframleidsla/LAN10201.px/.

[bib0054] Statistics Iceland (2024). https://px.hagstofa.is/pxis/pxweb/is/Atvinnuvegir/Atvinnuvegir__landbunadur__landbufe/LAN10103.px.

[bib0055] Swelum A.A., El-Saadony M.T., Abd El-Hack M.E., Abo Ghanima M.M., Shukry M., Alhotan R.A., Hussein E.O.S., Suliman G.M., Ba-Awadh H., Ammari A.A., Taha A.E., El-Tarabily K.A. (2021). Ammonia emissions in poultry houses and microbial nitrification as a promising reduction strategy. Sci. Total. Environ..

[bib0051] Simapro (2024). Ecoinvent LCI Database.

[bib0056] Turner I., Heidari D., Pelletier N. (2022). Life cycle assessment of contemporary Canadian egg production systems during the transition from conventional cage to alternative housing systems: update and analysis of trends and conditions. Res., Conserv. Recycl.,.

[bib0057] United Nations. (2022). *World Population Prospect 2022:summary of results*.UN DESA/POP/2022/TR/NO.3.

[bib0058] Usva K., Hietala S., Nousiainen J., Vorne V., Vieraankivi M.-L., Jallinoja M., Leinonen I. (2023). Environmental life cycle assessment of Finnish broiler chicken production – Focus on climate change and water scarcity impacts. J. Clean. Prod..

[bib0059] Van Dijk M., Morley T., Rau M.L., Saghai Y. (2021). A meta-analysis of projected global food demand and population at risk of hunger for the period 2010–2050. Nat. Food.

[bib61] Van Oers L., Guinée J. (2016). The abiotic depletion potential: background, updates, and future. Resources.

[bib0060] Willett W., Rockström J., Loken B., Springmann M., Lang T., Vermeulen S., Garnett T., Tilman D., DeClerck F., Wood A., Jonell M., Clark M., Gordon L.J., Fanzo J., Hawkes C., Zurayk R., Rivera J.A., De Vries W., Majele Sibanda L., Murray C.J.L. (2019). Food in the Anthropocene: the EAT–Lancet Commission on healthy diets from sustainable food systems. The Lancet.

