# Supplementary material for: A life cycle assessment of broiler chicken meat and egg production in Iceland
Source: Poult Sci. 2025 Mar 20;104(6):105072. doi: 10.1016/j.psj.2025.105072 (PMC12005881; doi:10.1016/j.psj.2025.105072)
Supplement: Supplementary file 1 [file mmc1.docx]

*Supplementary material*

*Calculations for Nitrogen Emissions from Manure Management.* To calculate direct and indirect Nitrogen (N_2_O) emissions that are generated by the birds in the form of manure, the following equations were used from the LEAP report, which uses equations from the 2006 IPCC Guidelines for National GHG Inventories (FAO, 2016)

Equation 1 Manure production for non-laying hen (FAO, 2016).

$$N_{E-PH}=\frac{{FI}_{PH}-C_{CP}}{6.25} \left( 1-NRF \right)=\frac{g N}{phase}$$

Equation 2 Manure production for laying hens not gaining body weight (FAO, 2016)

$$N_{E-PH}=\frac{FI.C_{CP}}{6.25} (\left( 0.0182 {EGG}_{wt} \right)\left( {EGG}_{prod} \right))=\frac{g N}{day}$$

Equation 3 Direct N2O emissions from manure management (FAO, 2016).

$$N_{2}O=N_{E}\left( {EF}_{MMS} \right)\left( \frac{44}{28} \right)=\frac{Kg N_{2}O}{day}$$

Equation 4 Volatile Solids (FAO, 2016).

$$VS={FI}_{PH}\left( 1-DMD \right)\left( 1-A \right)=\frac{Kg VS N}{phase}$$

The following equations are from the updated IPCC guidelines (2019) for Tier 1 calculations. They were used to estimate the indirect emissions and nitrogen leaching.

Equation 5 Nitrogen that is lost due to volatilization of NH_3_ and NO_x_, equation 10.26 (IPCC, 2019).


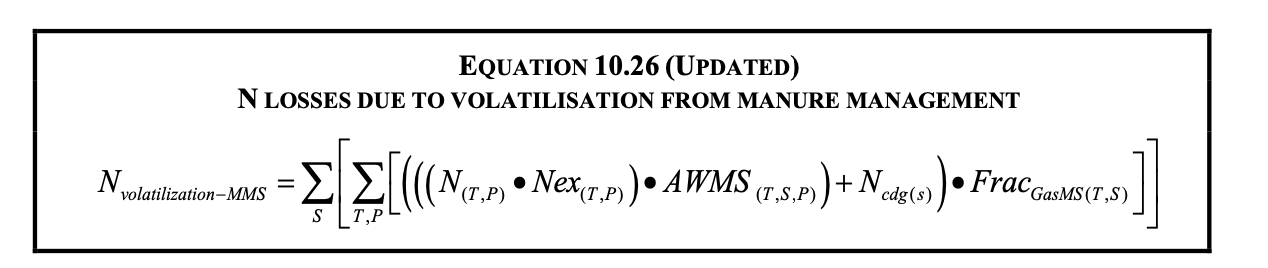


Equation 6 Amount of manure nitrogen that is lost due to leaching, equation 10.27 (IPCC, 2019).


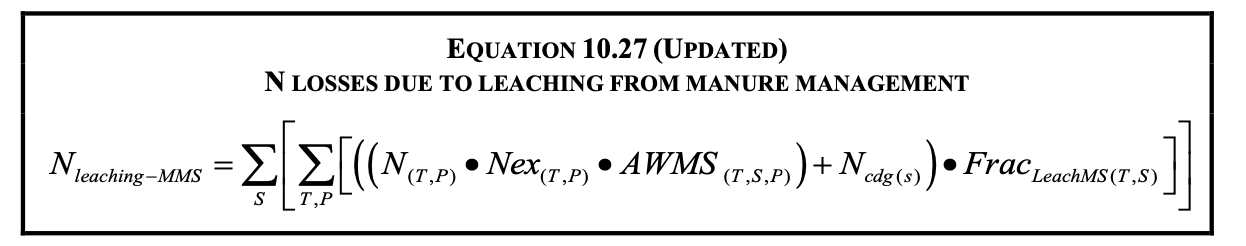


Equation 7 Indirect N_2_O emissions due to volatilization of N from manure management, equation 10.28 (IPCC, 2019)

$$N_{2}O_{G(mm)}=(N_{volatilization-MMS}\cdot{EF}_{4})\cdot\frac{44}{28}$$

Equation 8 Indirect N_2_O emissions due to leaching and runoff from manure management, equation 10.29 (IPCC, 2019)

$$N_{2}O_{L\left( mm \right)}=\left( N_{leaching-MMS}\cdot{EF}_{5} \right)\cdot\frac{44}{28}$$

***Calculations for Methane Emissions from Manure Management*** Methane (CH_4_) emissions caused by manure from broiler chickens, parent birds, pullets, and laying hens were calculated using the equation for volatile solid excretion (eq.4) and housing emissions for methane (FAO, 2016).

Equation 9 Housing emissions - Methane (FAO, 2016)

$${CH}_{4}=VS \left( B_{o} \right)\left( MCF \right)\left( \varrho\right)=\frac{Kg {CH}_{4}}{day}$$

Methane emissions from enteric fermentation were not considered following the IPCC (2019) guidelines, as it states that there is insufficient data for these calculations when it comes to poultry.

***Calculations for N2O emissions from Managed Soils*** To estimate the amount of Nitrogen available to be applied to soil from the manure, the following equations were used from IPCC chapters 10 and 11.

Equation 10 Fraction of Managed Manure N Lost Prior to Application to Managed soils for the Production of Feed, Fuel or for Construction uses, Equation 10.34A (IPCC, 2019)

$${FRAC}_{LOSS {MS}_{(T,S)}}={FRAC}_{GAS{MS}_{(T,S)}}+{FRAC}_{LEACHS{MS}_{(T,S)}}+{FRAC}_{N2{MS}_{(S)}}+{EF}_{3}$$

Equation 11 Estimation of FRAC _N2MS_, Equation 10.34B (IPCC, 2019).

$${FRAC}_{N2{MS}_{(S)}}=R_{N2 (N2O)}\cdot{EF}_{3 (S)}$$

Equation 12 Managed Manure N is available for application to managed soils, feed, fuel, or construction uses. Equation 10.34 (IPCC, 2019).


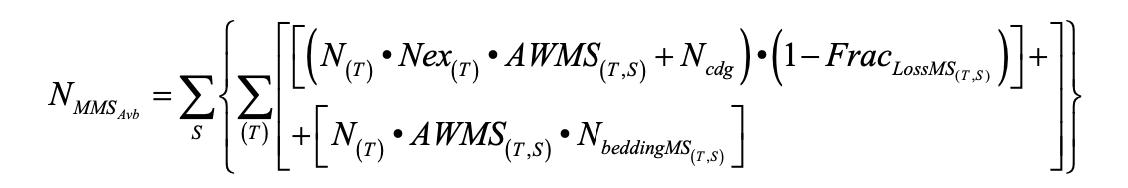


Equation 13 N from animal manure applied to soils, Equation 11.4 (IPCC, 2019).

$$F_{AM}=N_{MMS Avb}[1-\left( {Frac}_{FEED}+{Frac}_{FUEL}+{Frac}_{CNST} \right)]$$

Equation 14 Direct N2O emissions from managed soils, Equation 11.1 (IPCC, 2019)

$${N2O}_{Direct}-N=N2O-N_{Inputs}+N2O-N_{OS}+N2O-N_{PRP}$$
